# Methods to address poultry robustness and welfare issues through breeding and associated ethical considerations

**DOI:** 10.3389/fgene.2014.00407

**Published:** 2014-11-26

**Authors:** William M. Muir, Heng-Wei Cheng, Candace Croney

**Affiliations:** ^1^Department of Animal Sciences, Purdue UniversityWest Lafayette, IN, USA; ^2^Livestock Behavior Research Unit, United States Department of Agriculture – Agricultural Research ServiceWest Lafayette, IN, USA; ^3^Department of Comparative Pathobiology and Department of Animal Sciences, Purdue UniversityWest Lafayette, IN, USA

**Keywords:** genomic selection, multi-level selection, kin selection, ethics, behavior, animal welfare, indirect genetic effects, robustness

## Abstract

As consumers and society in general become more aware of ethical and moral dilemmas associated with intensive rearing systems, pressure is put on the animal and poultry industries to adopt alternative forms of housing. This presents challenges especially regarding managing competitive social interactions between animals. However, selective breeding programs are rapidly advancing, enhanced by both genomics and new quantitative genetic theory that offer potential solutions by improving adaptation of the bird to existing and proposed production environments. The outcomes of adaptation could lead to improvement of animal welfare by increasing fitness of the animal for the given environments, which might lead to increased contentment and decreased distress of birds in those systems. Genomic selection, based on dense genetic markers, will allow for more rapid improvement of traits that are expensive or difficult to measure, or have a low heritability, such as pecking, cannibalism, robustness, mortality, leg score, bone strength, disease resistance, and thus has the potential to address many poultry welfare concerns. Recently selection programs to include social effects, known as associative or indirect genetic effects (IGEs), have received much attention. Group, kin, multi-level, and multi-trait selection including IGEs have all been shown to be highly effective in reducing mortality while increasing productivity of poultry layers and reduce or eliminate the need for beak trimming. Multi-level selection was shown to increases robustness as indicated by the greater ability of birds to cope with stressors. Kin selection has been shown to be easy to implement and improve both productivity and animal well-being. Management practices and rearing conditions employed for domestic animal production will continue to change based on ethical and scientific results. However, the animal breeding tools necessary to provide an animal that is best adapted to these changing conditions are readily available and should be used, which will ultimately lead to the best possible outcomes for all impacted.

## INTRODUCTION

Consumers and society in general are becoming more aware of ethical and moral dilemmas associated with confined rearing systems (Swanson, 2007; Croney et al., 2012). Simultaneously, industry personnel are concerned about competitive social interactions that are inherent to less confined rearing production systems (Swanson, 1995; Rodenburg et al., 2008; Lay et al., 2011). Such interactions can result in injuries, stress, and mortalities. Unfortunately, there is also a concern that selection for increased productivity contributes to these welfare issues (Oltenacu, 2009; Rodenburg et al., 2010; Muir and Cheng, 2013). These concerns have brought about mandated or consumer driven changes in the way poultry and livestock can be managed (Croney and Millman, 2007; Croney, 2010) and raised issues for sustainability of the industry (Mench et al., 2011; Swanson et al., 2011). These mandates can limit selection programs in the interest of well-being or robustness of the animal (Blokhuis et al., 2007; Michel et al., 2007; Sorensen and Fraser, 2010). However, selective breeding programs are rapidly advancing, enhanced by both genomics and new quantitative genetic theory. The objective of this review is to examine the potential for new breeding programs to address these concerns.

## BEHAVIOR AND MANAGEMENT

Laying hens were domesticated several 1000 years ago. Early domesticated chickens lived in a small group in backyards, scratching and foraging food from the ground, performing heritable behaviors such as dust-bathing and pre-laying nesting, and returning to settle in the evening. Over the past several decades, the management and production systems for laying hens have undergone dramatic changes, with many groundbreaking scientific discoveries and technological advances, such as intense animal breeding programmers and mass-produced housing facilities. Farming practices were shifted from backyard farming to the modern intensified and specialized industries such as the poultry industry. See Eitan and Soller (2012) for review.

Several housing systems for laying hens have been developed in the modern egg industry: cage systems (conventional cage, furnished/enriched cage, and colony cage) and non-cage (alternative) systems (single tiered/floor housing, multi-tiered/aviaries with or without integrated nest boxes, and outdoor/free-range; LayWel, 2004a). The conventional (battery) cage system is the most common commercial housing facility for laying hens in the United States and in most non-EU countries. Typically, 5–9 hens, provided 412–438 cm^2^/hen, are housed together. The advantages of a conventional cage system, compared to other housing systems, in hen welfare are (1) a stable social hierarchy associated with the small group size; (2) low mortality; (3) low risk of damaging feather pecking and outbreaks of cannibalism; (4) cleaner eggs with low levels of parasitism; (5) improved bird health with low levels of infection, bumble foot, keel bone damage (deformation and fractures), and aerial pollution; (6) low risk of predation; (7) easy management and care; and (8) high economic efficiency. However, there is considerable morphological, physiological, and behavioral evidence demonstrating that the use of battery cages increases stress reactions in hens and decreases quality of life due to an overcrowded and barren environment. The main disadvantages are (1) discomfort and abnormal behavior resulting from limited space for hens to perform heritable behaviors such as dust-bathing, roosting, and pre-laying nesting; (2) decreased bone quality (osteoporosis) with high susceptibility of fractures on depopulation; and (3) increased body injury from feather pecking and cannibalism, resulting from insufficient space to escape from dominants (LayWel, 2004a,b; Savory, 2004; Lay et al., 2011; Sumner et al., 2011). Given these problems, there is growing pressure from animal welfare and consumer groups advocating a global ban of battery cage systems in the poultry industry. Similar lobbing by organizations within Europe led to a ban on battery cages as of 2012 and only furnished cages and non-cage systems are allowed (CEC, 1999). Poultry producers and scientists are in a prime position to develop hen-friendly housing systems that minimize stress and safeguard welfare while maintaining the favorable characteristics previously found in cage-based production system.

Various furnished (also called enriched) cage systems have been developed to meet the hen’s behavioral repertoire including: large group cages housing 60 or more hens; medium group cages for 15–30 hens; and small group cages for up to 15 hens (LayWel, 2004a). Furnished cages attempt to provide enrichment to hens while still taking advantage of the benefits of a small group size. The cages are equipped with perches, dust baths, and nesting areas allowing for the hens to meet “the needs for their natural behaviors,” such as nesting, roosting, and scratching (Appleby, 1998; Newberry, 1999; Cordiner and Savory, 2001; Appleby et al., 2002). Previous studies have shown that birds housed in furnished cages also experience improved well-being due to reduced fear, aggression, and feather pecking, and increasing bone mineral density (Newberry, 1995; Vits et al., 2005). Although furnished cage system seem to be a possible way to improve hen welfare, high mortality and feather pecking and cannibalism can occur, particularly with non-beak trimmed hens; additionally, bumble foot and keel bone damage can result from roosting (Vits et al., 2005; Sandiland et al., 2009).

Several non-cage (alternative) systems have also been developed for hens to express more of their behavioral repertoires, especially foraging, dust-bathing, and nesting; with freedom to display wing-flapping and flying. The most popular are single tiered/floor housing; multi-tiered/aviaries with or without integrated nest boxes; and outdoor/free range system. These housing systems are becoming more commonplace, especially in European countries. The main disadvantages of non-cage systems, compared to cage systems, are: (1) unstable hierarchy associated with the large flock group size; (2) high levels of mortality resulting from high risks of feather pecking and cannibalism; (3) high risk of hens sustaining fractures associated with collision damage with perches, nest-boxes, and other structures; (4) increased risk of smothering; (5) increased risk of disease and parasites due to contact with droppings, infective agents, and wild birds; (6) increased risk from predation; and (7) reduced egg production due to high mortality and poor bird welfare, especially in subordinate hens which may have limited access to feed, water, and other provided structures (nest-boxes and range) due to aggressive encounters and resource guarding by dominant hens (LayWel, 2004b; Lay et al., 2011). Further studies are needed to investigate optimal housing designs to improve access to food and water such that welfare of subordiante birds is imporoved, such as dividers, distribution, and means for alternative access.

Each housing system has itself advantages and disadvantages for the welfare of laying hens realtive to the five freedoms (Shimmura et al., 2010, 2011; Huneau-Salaun et al., 2011; Lay et al., 2011; Mench et al., 2011; Tuyttens et al., 2011). Its influences are hen strain-, age-, and facility-dependent. Furnished cage systems and non-cage (alternative) systems are developed for hens to express their nature behaviors. However, there is a high risk of reduced stability of hen social hierarchies and poorer welfare on a flock basis in all systems, i.e., a large group size with high risk of feather pecking and cannibalism. Feather pecking and cannibalism are significant contributors to mortality rates chickens with untrimmed beaks. Beak trimming is a common practice to prevent feather pecking and cannibalism. However, beak trimming causes tissue damage, exposing billions of chickens to pain (acute, chronic, or both) annually. Beak trimming is not an acceptable intervention to prevent feather pecking and mortalities but genetic selection may provide opportunities to reduce the need for beak trimming.

## GENETIC SELECTION AND IMPACTS ON WELL-BEING

“Should we change housing to better accommodate the animal or change the animal to accommodate the housing” is a rather old question facing new changes especially concerning welfare of laying hens housed in modern intensive production systems (Cheng, 2007). Recent research findings have indicated that an animal’s welfare is dependent on its genetic characteristics, environmental factors, and genetic–environmental interactions, i.e., an animal has the ability to adapt to its environment and the environment leads to behavioral and physiological plasticity in the animal. The outcomes of adaptation could lead to improvement of animal welfare by increasing fitness of the animal for the given environments, which might lead to increased contentment in those systems. Genetic selection for phenotypic characteristics associated with specific physiological or behavioral displays, including domestic behavior, has become a major tool to improve animal production and welfare. Studies have evidenced that animal productivity and welfare can be improved at the same time through genetic selection (Muir, 1996; Cheng and Muir, 2005; Cheng, 2010a). Genetic improvements of farm animals, with the discovery of genomic sequences, may speed up breeding programs and has the potential to be used very successfully in selecting laying hens with high production efficiency and optimal welfare, resulting from resistance to stress, disease or both. Primary among new selection methods are (1) multi-level and multi-trait selection directed at improving associative effects and (2) genomic selection (GS).

### SELECTION PROGRAMS TO IMPROVE ASSOCIATIVE EFFECTS

In production environments social interactions are ubiquitous and unavoidable, except by housing animals individually, which is neither practical nor desirable as isolation is itself a stressor. Associative effects are social impacts of one animal on the performance of another (Muir and Schinckel, 2002). Such impacts can be positive, such as with mutualism where stress is abated by companionship, or antagonistic, such as with pecking and cannibalism. When such behaviors are inherited, the environmental effect on the target animal is a genetic effect in the associated animal. These inherited social effects were first defined as associative effects (Griffing, 1968, 1977) and later as indirect genetic effects (IGEs; Agrawal et al., 2001; Bijma and Wade, 2008). There are three methods to improve associative effects, i.e., either reduce negative or increase positive IGEs: (1) direct selection to reduce aggressiveness, such as pecking (Kuo et al., 1991; Kjaer and Sorensen, 1997; Kjaer et al., 2001), (2) multi-level selection (Bijma and Wade, 2008; Wade et al., 2010a; Muir et al., 2013), and (3) multi-trait selection where the direct and associative effects of each animal are estimated and directly selected for in an index (Muir and Schinckel, 2002; Muir, 2005; Bijma et al., 2007a,b).

#### Multilevel selection

Multilevel selection theory focuses on merit relative to levels of organization, i.e., groups and within group. This concept was originally developed in the context of non-interacting genotypes, i.e., no social effects (Lush, 1947, 1971). The issue was how much weight to place on the family means as opposed to individuals within family. Lush developed an optimal index for weighting the independent sources of variation which was purely a function of heritability. As the heritability decreases the weight on the family mean increases to average out environmental effects. At the opposite extreme, with high heritability, most of the weight is placed on individual merit because there are no (or minor) environmental effects to average out. In this regard, housing was a side issue, animals housed individually were treated the same as animals housed in groups. In the next 20 years Henderson and Quaas (1976) and Henderson (1984a,b) developed mixed models and BLUP which replaced the Lush index as it always produces the optimal weights on individual vs. family means assuming individual performances are independent, i.e., no social effects.

Griffing (1967, 1977) extended Lush’s concept to include interacting genotypes, including social effects. With interacting genotypes it was necessary to define a new trait; that trait was the social effect of one animal on another and was called the associative effect, in contrast to genes that have effects on the animal’s own performance, which were called direct effects. The associative effect is an IGE of one animal on another and later termed IGE’s (Wolf et al., 1998; Agrawal et al., 2001). Griffing showed that if the direct and IGE effects are negatively correlated, then individual selection would be antagonistic to selection goals and actually increase negative social interactions, i.e., a gene increases performance of the individual, but has negative impacts on the trait to others in the group. In this situation Griffing recommended “group selection” where groups consist of related individuals, i.e., families. Theoretically, group selection always improves group adaptations regardless of the sign of the genetic correlation. Group selection is an extreme form of multi-level selection where all the weight is placed on the family mean.

**FIGURE 1:**
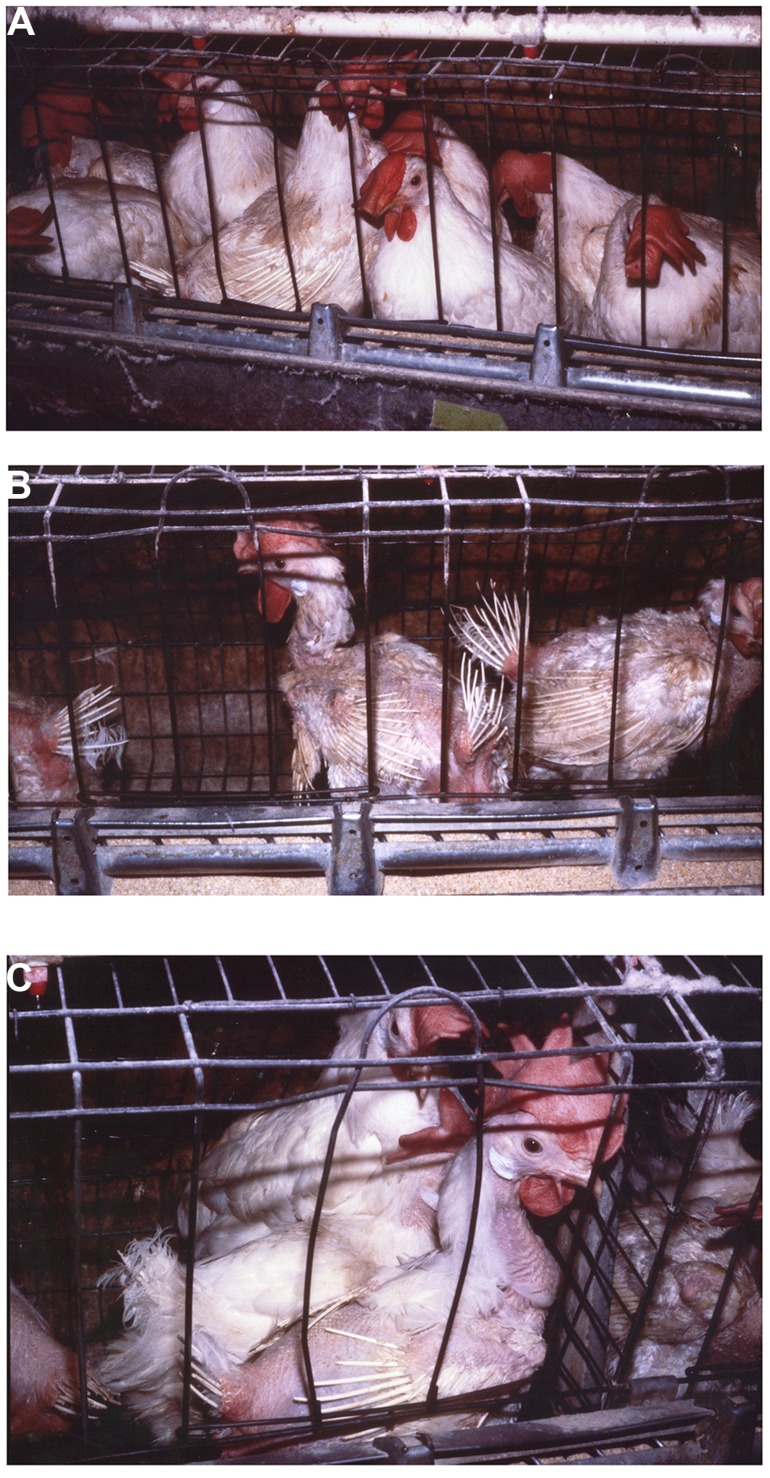
**KGB (A) and DXL (B,C) chickens after 12 months of housing in 12 bird colony cages**.

Group selection has been shown to be highly effective in improving productivity while also improving animal well-being (Craig and Muir, 1996a,b; Muir, 1996; Cheng et al., 2001a,b, 2002, 2003; Cheng and Muir, 2005; Bolhuis et al., 2009; Rodenburg et al., 2010; Wade et al., 2010b; Nordquist et al., 2011; Kops et al., 2013; Nicol et al., 2013). Muir (1996) was the first to apply group selection in domesticated animals. In that experiment, a sample of the commercial Dekalb Delta X layer line (Dekalb Poultry Reserch, Dekalb Ill) was obtained. A random bred line from the same stock was maintained as a control (C). The group selected chicken we termed the Kinder Gentler Bird (KGB). Craig and Muir (1996a,b) and Muir (1996) showed that annual percentage mortality of the group selected line in multiple-bird cages without beak trimming decreased from 68 to 8.8% in five generations while eggs per hen housed increased from 91 to 237. Mortality in group housed birds at the termination of the experiment was no different than that in single bird cages demonstrating that mortality due to competitive interactions had been greatly reduced or eliminated. **Figure 1** clearly demonstrates improvement in feathering and survival associated with reduced pecking and negative social interactions in group selected birds (KGB) (**Figure 1A**) as opposed to individual selection (DXL) (**Figures 1B,C**). Physiological indicators showed that group selection caused changes in behavior, stress physiology, and immunology (Cheng et al., 2001a,b; Cheng and Muir, 2004). Interestingly, group selection also had impacts on robustness, a trait not associated with social interactions. In multiple-hen cages the KGB had an increased resistance to heat exposure, as indicated by lower mortality, when compared to the control and commercial lines (Hester et al., 1996) indicating the group selected KGB birds had an overall greater ability to cope with stressors.

The data also supported the conclusion that individual selection in non-social environments can worsen animal well-being and performance in social environments (Craig and Muir, 1996a,b). In that experiment a second sample of the Dekalb line was taken 20 years after the first. The Dekalb line had continued under commercial development and was selected based on productivity in single bird cages using essentially a Lush (1947) “optional” index, and later updated to traditional animal model BLUP (Harris and Newman, 1994). The new sample of the Dekalb Delta was designated (X). These two Dekalb lines (X and C) were compared to each other and the KGB for production and mortality in both single and 12 bird colony cages. Housing was at 17 weeks of age, and without beak trimming. Results showed that in single-bird cages, in terms of eggs per hen housed, eggs per hen per day, egg weight and egg mass, all were significantly greater for X than for the KGB line, which in turn was greater than C. However, in 12-bird cages the reverse was seen, with the KGB line superior to X and C for eggs per hen housed, egg mass, and eggs per hen per day. The most remarkable difference was for mortality. The X line experienced 89% mortality at 58 weeks of age as compared to the group selected KGB line with 20% and C at 54%. Clearly continued individual selection of X, as compared to its original performance (C), caused further deterioration of well-being in social situations. In contrast, group selection almost eliminated the problem. It should be noted that the experiments were conducted without beak trimming and with full light such that the full extent of the behavior could manifest itself. In commercial production environments, birds are beak trimmed and lights dimmed to control cannibalism due to pecking.

A less extreme form of multi-level selection is sometimes called kin selection, but the literature is inconsistent in this regard (Wade et al., 2010a). This form of selection is based on performance of the individual where animals are housed in family groups. Because individual performance is affected by all of the individuals in the group, if those individuals are related, the performance of the individual automatically includes its own associative effect. Thus individual selection in kin group should improve both direct and IGE effects. This theory was tested by Muir et al. (2013). In that experiment, Japanese quail (*Coturnix japonica*) were used as a model. The quail were housed in 16 bird colony cages and selected for increased 43 days weight. A positive control was also utilized in which identical selection procedures and models were used (animal model BLUP). The only difference in selection methods was the way in which quail were allocated to cages. The control was allocated at random whereas kin were allocated by half-sib family.

The results showed that responses to selection for increased 43 days weight using kin selection were an order of magnitude greater than for the control. Response of the control was not significantly different from zero. Overall mortality due to fighting and cannibalism for Kin and Random grouping was respectively 6.6 and 8.5%, the difference was highly significant. Mortality was a correlated trait responding as an IGE, as expected with multi-level selection. Thus multilevel selection in kin groups was effective in reducing detrimental social interactions while improving productivity.

#### Multi-trait selection

Neither group selection nor kin selection as practiced above is optimal. The optimal multi-level selection program is dependent on correctly weighting the family vs. individual merit. These weights are dependent on a number of factors, including, the genetic variances for direct and IGE effects, the correlation between them, the group size, and the degree of relationship within groups (Griffing, 1977; Bijma et al., 2007a,b; Bijma and Wade, 2008; Bijma, 2010b). An alternative is multi-trait selection. Muir and Schinckel (2002), Muir (2005), and Bijma et al. (2007a,b) extended the theoretical results of Griffing (1977) to a multi-trait mixed model such that the direct and IGE effects, and their (co)variances, could be estimated. In a companion to the Muir et al. (2013) experiment with quail, Muir and Schinckel (2002) and Muir (2005) had also performed optimal two-trait selection for direct and IGE effects, the optimal being one times the direct effect and n-1 times the associative, and randomly assigned to cages, the same as the control (C).

Although the selection program was effective, and much more so than the control, the two-trait approach did not achieve the theoretical gains expected and was most likely due to errors in the genetic (co)variances, and as with any multi-trait selection program, selection induces changes in the genetic parameters, making construction of an optimal index problematic. Estimation of the genetic parameters for direct and associative effects requires moderately large data sets of groups of a small numbers of families (Bijma, 2010a). Moreover, the use of an optimal index requires recording of individual phenotypes within group, which may be difficult for egg production. Nevertheless, the optimal breeding program, even with the two-trait, is to rear animals in kin groups. In this way accuracy of selection for total breeding value is maximized (Ellen et al., 2007).

#### Implications of selection for associative effects on breeding programs and management

Because feather pecking can be effectively addressed by group selection, the need for beak trimming as a management practice is greatly reduced or eliminated. Also group selection increases robustness as indicated by the overall greater ability to cope with stressors suggesting that group selection is an effective method to increase robustness which should impact management. In terms of sustainability of domestication breeding programs, kin selection is easy to implement, does not require multi-trait estimates of genetic parameters and is thus robust to parameter estimation errors (Grundy et al., 1994), and is expected to improve both productivity and animal well-being similar to group selection, but with somewhat lower levels of inbreeding because families are not the unit of selection as with group selection. On the other hand, kin selection, and two-trait selection require assessment of productivity on an individual level which can be problematic for layers where group housing often makes individual records difficult except with trap nesting.

Because associative effects are improved with multi-level selection, it might be possible to increase stocking density, increase light levels, use larger groups in floor pens, and with increase in robustness, production animals may not be as sensitive to environmental stressors and disease. However, ethical concerns also limit those choices. Just because the animal can now cope better with more intensive agriculture environments should not be used as justification to allow extreme conditions. Ethical consideration need to be considered. Two other issues that need to be further researched are (1) GxE interactions, i.e., will selection to improve social effects in one environment, such as battery cages, improve social effects in another, such as floor pens. And (2) Cross breeding programs. Commercial production is often on crosses between lines, while selection is within line. In the case of hybrids, is heterosis for social effects positive? Some preliminary observational data in a commercial floor pen setting with the KGB birds suggests that social effects were also improved in large floor pens. Further testing of the KGB (C. Danchin, personal communication, October 12, 1998) showed that when the KGB was crossed to a commercial pure bred layer, mortality and aggression was more like the commercial bird than the KGB, suggesting that heterosis for social effects is for individual and not group performance. This result would imply that if crossbreds are used, that selection must be for crossbred social effects, or that both lines need to be group selected for improved social effects.

### TRAITS OF SELECTION AND SELECTION METHODS

Direct selection against traits associated with unwanted behaviors, is effective but requires measuring behavior on 100–1000s of animals to implement and raises practical implementation issues (D’Eath et al., 2010). More easily measured proxy traits can be used if shown to be highly related to the behavioral trait. However, direct selection on either behavior or physiological objectives should be viewed with caution. The intended results may not be as expected. For example, Webster and Hurnik (1991) showed that traits associated with non-aggression, such as sitting and resting, were negatively correlated with productivity. Furthermore, the link between behavior and stress is misinterpreted. For example, Duncan (1979) showed that a flighty strain of birds which exhibited avoidance and panic behavior following stimulation returned to a normal heart beat sooner than a line of more docile birds, implying that docile birds may be too frightened to move. Similar problems can occur if selection is directed at the physiological responses to stress. Gross and Siegel (1985) were successful in selecting lines of birds for high and low plasma corticosterone in response to social strife but further testing (Siegel, 1993) showed that the birds did not differ in their corticosterone response to a non-social stressor. Thus, direct selection on specific behavior traits may not lead to improved animal welfare overall.

Selection response may be enhanced by GS. GS is a relatively new selection method based on genome wide predicted breeding values (GEBVs), which was first proposed by Meuwissen et al. (2001). This selection method coincides with the new single nucleotide polymorphism (SNP) technology which is high throughput, accurate, and relatively inexpensive. The concept of GS is to estimate effects of all markers simultaneously in a random effects mixed model. GS requires dense markers spaced across the genome (equal spacing being optimal without prior knowledge of QTL positions), thereby taking advantage of all available genetic variation in population wide linkage disequilibrium (LD) with those markers. Many different evolutions of GS has since evolved based on alternative assumptions and methods to estimate effects (Fernando et al., 2007; Calus et al., 2008; Aguilar et al., 2010, 2011b; Calus, 2010; Hayes and Goddard, 2010; Calus and Veerkamp, 2011; Habier et al., 2011, 2013; Meuwissen et al., 2011, 2013; VanRaden et al., 2011; Christensen et al., 2012; Garrick et al., 2014). GS has the potential to change the structure of genetic improvement schemes. For example, there are a number of traits that cannot be directly recorded on the selection candidates, e.g., performance under crossbreeding conditions, laying performance in males, slaughter quality, disease resistance, and social interaction traits. Moreover, with traditional BLUP evaluation of breeding values based on sib information, such as egg production and disease resistance, phenotypic BLUP cannot differentiate among full sibs, while this is theoretically possible with GS (Goddard et al., 2010; Garrick, 2011; Daetwyler et al., 2012; Goddard, 2012; Calus et al., 2013; Chen et al., 2013). GS also provides the opportunity to select for such traits at a younger age, i.e., genotyping and selection can occur at hatching.

The potential of GS was demonstrated by Muir (2007) using simulations based on associations with single SNPs. Relatively, GS more than doubled the accuracy of selection for a trait of low heritability (0.72 vs. 0.32). These results suggest that with adequate numbers of phenotypes and sufficiently dense SNP chip, response with GS can exceed traditional BLUP, but especially for traits of low heritability. This results because for traits of high heritability, additional information from genomics, or other sources, cannot improve accuracy. In practice GS may not increase the accuracy of selection for a number of reasons, including: (1)The density of the SNP chip is not adequate; (2) LD structure of the species is not favorable, with large LD blocks SNP effects are confounded; (3) Number of samples in the training population is low (Goddard et al., 2010). For low heritability traits, more phenotypic data is needed to train the model than with high, thus there is a trade off in terms of time and resources; And (4) the proportion of the genetic variation that is additive is small. A trait with low heritability can result because the trait has high non-additive genetic variation rather than high environmental variation. In which case neither progeny testing or GS can increase the accuracy of selection, unless some cross breeding program is considered. As a result, the success of GS is likely to vary greatly depending on the trait, species, SNP chip, and amount of phenotypic data collected.

There are several other limitations of GS, including additional cost (Tribout et al., 2012, 2013; Abell et al., 2014) and the need to control inbreeding (Goddard et al., 2010). Sonesson et al. (2012) concluded that to control inbreeding, “it is necessary to account for it on the same basis as what is used to estimate breeding values, i.e., pedigree-based inbreeding control with traditional pedigree-based BLUP estimated breeding values and genome-based inbreeding control with genome-based estimated breeding values.” Simulation studies have also shown that if inbreeding rates are constrained for optimal contributions of breeding animals, that improvements of GS would be significantly reduced (Lillehammer et al., 2011; Bouquet and Juga, 2013). Finally several simulations have shown that the accuracy of GS rapidly declines rapidly after selection starts (Muir, 2007; Sonesson and Meuwissen, 2009; Bastiaansen et al., 2012) necessitating the continued collection of phenotypes to update the models.

Genomic selection is currently undergoing testing in many species (Legarra et al., 2008; Hayes et al., 2009a,b; VanRaden et al., 2009; Chen et al., 2011, 2013; de Roos et al., 2011; Wolc et al., 2011; Duchemin et al., 2012; Gao et al., 2012; Su et al., 2012; Azevedo et al., 2013; Bolormaa et al., 2013; Carillier et al., 2013; Colombani et al, 2013; Ding et al., 2013; Lillehammer et al., 2013; Badke et al., 2014; Baloche et al., 2014; Boddhireddy et al., 2014; Nordbø et al., 2014) with mixed results. Differences in methods to implemnent GS, SNP density, species, LD, traits of selection, and number of traits selected make comparisons difficult. Also, most studies compare accuracy based on a single generation of data, few studies reported actual multi-generation selection results and even fewer studies have directly comparted GS to phenotypic BLUP in a multigenerationl selection experiment. A noteable exception was a commercial test by a layer breeding company (Hendrix Genetics) using a 60 K SNP chip (Heidaritabar et al., 2014). Traditional BLUP and GS selection methods were compared side by side in three different lines of egg-laying chicken. For all lines, the responses for GS over BLUP were between 21 and 62% depending on line. However, the greatest impact of GS was annual rate of progress is due to shorter generation intervals. With traditional BLUP 2 years were required per generation of selection. With GS, selection is possible at the hatch, with breeding occurring 20 weeks later, or two generations per year. Thus on an annualized basis, the rate of progress was increased a minimum of 400% and with the increase in accuracy factored in, the rate of improvement is between 500 and 600%. The economic impact of which is large considering that one pure line breeder is multiplied in 550,000 commercial birds. However, with the increased turn over of generations, the rate of inbreeding per unit of time increases, which will limit future progress (Robertson, 1960; Hill, 1985), ability to meet changing objectives or challenges (Muir et al., 2008) and negatively affect fitness and animal wellbeing (AWB) (Meuwissen and Woolliams, 1994; Hedrick and Kalinowski, 2000; Goddard, 2009).

#### Selection programs, limitations, ethical considerations, and animal well-being

Animal well-being traits are often expensive or difficult to measure, or have a low heritability, such as pecking (Kjaer and Hocking, 2004; Buitenhuis and Kjaer, 2008), cannibalism (Kjaer and Hocking, 2004), robustness (de Jong and Bijma, 2002; Kanis et al., 2004; Mulder et al., 2009), mortality, leg score, bone strength (Hocking, 2010), disease resistance (Cheng et al., 2008; Cheng, 2010b), and pulmonary hypertension in broilers (Emmerson, 1997; Julian, 1998; Hocking, 2010). These traits may respond more rapidly to GS provided the economics, LD structure, and genetic architecture are favorable as discussed previously. However, GS cannot overcome issues that are inherent with collecting phenotypes, i.e., accuracies of recording and trait definitions. Phonemics and the accurate definition of traits and how to measure them may be the next challenge for breeders (Houle et al., 2010).

As an example, Mark and Sandoe (2010) discussed potential impacts of GS on dairy cattle breeding for the welfare of dairy cows. They note that in the past, some emphasis has been placed on rather poorly defined measures of traits relevant to cow welfare, including calving ease score and ‘clinical disease or not’ but such selection has not been sufficient to overcome these issues given the current unfavorable genetic trends for metabolic, reproductive, claw and leg diseases in dairy cattle. The authors expressed concern that GS may facilitate breeding schemes that reduce generation intervals and carry higher risks of unwanted side-effects on animal welfare. They advocate a need for measuring traits related to animal welfare and include selection pressure on those traits, either through GS or traditional breeding.

Similarly, in poultry, animal well-being traits are generally poorly defined and rarely measured. In broilers, production traits, such as growth, may need to be de-emphasized due to possible conflicts with robustness (Rauw et al., 1998; Knap, 2005, 2012). Robustness is the ability to combine a high production potential with resilience to stressors, allowing for unproblematic expression of a high production potential in a wide variety of environmental conditions (Knap, 2005, 2012). Robustness may be reduced when production-related processes demand so many resources that coping and immune responses are compromised (Knap, 2005, 2012). Other traits related to AWB need to be collected to at least ensure AWB is not being compromised as a result of the traits being selected.

All of these concerns emphasis the need to relate traits of selection, and the selection program itself, to animal well-being. As detailed previously, selection on behavior traits may not improve AWB and selection on production traits may compromise AWB, emphasizing the need for a comprehensive breeding program where traits are well-defined, recorded, and combined with a breeding program/objective that directly includes AWB. The only breeding programs that ensures that AWB will improve while at the same time improves production traits are multi-level selection and multi-trait selection where one of the traits includes IGEs. Multi-level selection emphasizes productivity of the group, not the individual, while multi-trait selection including IGEs is an alternative method to achieve the same goal. Both of these programs can include possible enhancement from GS. GS can be combined with multi-level selection and multi-trait IGE selection to IGEs and animal well-being. The methodology is strait forward using the single step method (Legarra et al., 2009; Aguilar et al., 2010, 2011a; Chen et al., 2011; Christensen et al., 2012; Legarra and Ducrocq, 2012). With the single step method of GS, the genomic information is integrated into the pedigree relationship matrix. For BLUP evaluation, either in a multi-level evaluation setting, or in a multi-trait evaluation including IGEs, the augmented relationship matrix is used directly in the mixed model equations to derive BLUP estimates of either direct effects or IGEs. In this way, GS can increase the response in production traits while at the same time improve AWB through improved social effects measured as IGEs.

A major citicism of modern food animal production is the failure to adequately consider the ethical implications of current and proposed pratices, including genetic selection of animals. Although the criteria and methods used for selection are often well-described relative to their scientific implications, the broader ethical issues that are embedded tend to be poorly addressed. All of these areas must be well-understood to ensure that sound decisions are made. It is well-established that although science can help to gage the risks of decisions, science cannot decide what level of risk is acceptable to all whom are impacted (Mench, 2003). The latter question falls squarely in the realm of ethics (Croney et al., 2012).

The criteria for trait selection must be scrutinized. Those giving due consideration to the impacts of selection on animals must consider to what extent the selection of certain traits at the expense of others is in the subject animals’ best interests. For example, as noted previously, selecting animals that fit the environments in which they are kept may appear to resolve ethical concerns in regard to keep them in environments that do not fully meet their welfare needs. Following that logic, given that laying hens cannot perform certain key behaviors such as dust-bathing and nestbuilding in many commercial operations and that this compromises their well-being, it might seem reasonable to consider selecting for birds that do not (apparently need to) express such behaviors. However, while this may resolve one scientific concern, it may raise others and accompanying ethical concerns. For example, lack of expression of a behavior is not de facto evidence of lack of motivation to do so. Motivation to perform dust-bathing behavior may still exist although the actual performance of or threshold for stimulating the behavior may be altered. Further, even if the question of motivation can be resolved, behaviors such as dust-bathing serve a functional purpose (control of ectoparasites) that can be impaired by altering expression of that behavior. Consequently, selecting for reduced or eliminated dust-bathing may make economic sense (eliminating the need to provide dust baths to hens and loss of energy expended in dust-bathing) and may appear at first glance to resolve a public concern. However, it is certainly not in the best interest of the hen who still must cope with parasites, but now has one less means by which to do so.

Moreover, the idea that dust-bathing is a fundamental component of hen ethology and that attempting to eliminate it (or similar behaviors) may negatively impact the telos (Rollin, 1995) or integrity of the species must be considered (Thompson, 2008) Although the concept of species integrity is in itself somewhat contentious (Sandøe and Holtug, 1998), here, the previous discussion of robustness becomes especially relevant. Determining which selection methods are ‘good’ or ‘best’ will depend in large part on their capacity to simultaneously attend to hens’ health and well-being as a function of the environments in which they are intended to be kept, while also addressing concerns related to species integrity (Star et al., 2008). The extent to which genetic integrity and animal welfare can be balanced should therefore be factored into selective breeding methods and decisions (Sandoe et al., 1999).

Given growing public concerns about intensification of animal agriculture (Swanson, 2007; Croney et al., 2012), especially relative to negative implications for animal welfare, attempting to select animals to fit increasingly more challenging or restrictive environments is likely to elicit public criticism. To avoid worsening existing problems, it is imperative that those with the authority to make breeding decisions focus not just on immediate concerns and a few traits of economic importance. Long-term impacts relating to various aspects of animal health and well-being must be appropriately considered. As is the case for all aspects of animal production, the selection methods used for layer hen production should be subject to ethical assessment as well as scientific scrutiny to ensure the best possible outcomes for all impacted.

## Conflict of Interest Statement

The authors declare that the research was conducted in the absence of any commercial or financial relationships that could be construed as a potential conflict of interest.
